# Methodological lessons in neurophenomenology: Review of a baseline study and recommendations for research approaches

**DOI:** 10.3389/fnhum.2013.00608

**Published:** 2013-10-10

**Authors:** Patricia Bockelman, Lauren Reinerman-Jones, Shaun Gallagher

**Affiliations:** ^1^Applied Cognition and Training in Virtual Environments Lab, Institute for Simulation and Training, University of Central FloridaOrland, FL, USA; ^2^Department of Philosophy, University of MemphisMemphis, TN, USA; ^3^School of Humanities, University of HertfordshireHatfield Hertfordshire, UK

**Keywords:** neurophenomenology, EEG, experience, simulation, experimental methods, phenomenological interview

## Abstract

Neurophenomenological (NP) methods integrate objective and subjective data in ways that retain the statistical power of established disciplines (like cognitive science) while embracing the value of first-person reports of experience. The present paper positions neurophenomenology as an approach that pulls from traditions of cognitive science but includes techniques that are challenging for cognitive science in some ways. A baseline study is reviewed for “lessons learned,” that is, the potential methodological improvements that will support advancements in understanding consciousness and cognition using neurophenomenology. These improvements, we suggest, include (1) addressing issues of interdisciplinarity by purposefully and systematically creating and maintaining shared mental models among research team members; (2) making sure that NP experiments include high standards of experimental design and execution to achieve variable control, reliability, generalizability, and replication of results; and (3) conceiving of phenomenological interview techniques as placing the impetus on the interviewer in interaction with the experimental subject.

## INTRODUCTION

The tension of scientific exploration involves both push and pull; it launches outward, across seas or solar systems, and yet draws us inward, into the depths of Earth and of our own minds. The topics presented in this article balance on that tension, with reference to a study of the inner experience of the mind as it faces outward toward the expanse of space. More specifically, the aim is to critically evaluate neurophenomenological (NP) methods for studying first-person experiences. We describe the techniques used in a baseline study and highlight areas for methodological improvement that may be helpful in future NP research. The baseline study acts as a reference point from which recommendations for methodological improvements may be extrapolated, contributing to the refinement of neurophenomenology as an approach for studying human experience.

After reviewing some of the context of the broader NP tradition, emphasizing its relationship to (and contrast from) other cognitive science methods (see Neurophenomenological Methodology), we present an overview of the baseline study (see Overview of the Baseline Experiment). The final section includes an outline of the key “lessons learned” about NP methodology in the baseline study.

## NEUROPHENOMENOLOGICAL METHODOLOGY

While the nature of human experience has been questioned since the time of Aristotle, there is still no consensus in the scientific community regarding methods to approach such inquiries. Neurophenomenology is one promising approach, as its core purpose is to connect research in the various fields that study experience in a manner that fully integrates first-person experiential accounts with third-person neuroscientific measurements. To achieve this goal neurophenomenology uses techniques from cognitive science, neuroscience, and philosophical phenomenology in an attempt to piece together a dynamic and inclusive picture.

As its name implies, neurophenomenology blends data collection methods from neuroscience and phenomenology. Phenomenology is a diversified philosophical approach initiated in the twentieth century by Edmund Husserl. It was conceived as a descriptive and analytic study of consciousness, including aspects of intentionality (the directedness or “aboutness” feature of consciousness) and phenomenality (the qualitative feel of what it is like to experience something). Philosophers in this philosophical tradition, like Heidegger (1962) and Merleau-Ponty (2012), emphasized pragmatic, existential, and embodied aspects of being-in-the-world and situated cognition. The neurobiologist, Varela (1996), built on the work of these philosophers in order to define the NP method.

One of the objectives of experimental neurophenomenology is to bridge first-person experience and neurophysiological data (Varela, 1996). As a form of naturalized phenomenology (Petitot, 1999) it introduces the phenomenological method into the experimental lab. In experiments by Lutz et al. (2002), for example, Varela and his colleagues used phenomenologically trained subjects to report on their experiences of readiness or unreadiness for a perceptual task. A set of experiential categories (“phenomenological clusters”) were generated from the reports of the participants in an initial training and testing session. Experimenters were then able to use these categories in subsequent testing to find strict correlations between dynamical brain processes (measured with electroencephalography, EEG), reaction times and the specific categories of experience.

Lutz et al. (2002), using NP methods, were able to capture and categorize variable attentional factors that are difficult to manipulate, though they enter into many empirical studies of perception, but are usually discounted in standard neuroscientific and behavioral studies using statistical averaging techniques – i.e., such factors are usually treated as “noise.” NP methods allowed the experimenters to show that specific variations in the patterns of endogenous synchrony in frontal electrodes just prior to presentation of a perceptual stimulus correlated precisely (and with stability over several recordings) with degrees of attention reported by the participants, and with behavioral performance (reaction times). The correlation with behavioral data may suggest that the same attentional variations could be defined in strictly behavioral terms. Use of behavioral measures alone, however, does not rule out the possibility that reaction time variations were due to other, non-attentional factors. Use of qualitative first-person reports further provides a richer and more precise characterization of the texture and structure of conscious experience. As Lutz et al. (2002) suggest, the NP strategy can be understood as an extension of traditional procedures in cognitive science based on the use of verbal reports and post-experiment questionnaires. The emphasis on first-person experience, and the use of detailed phenomenological methods by trained subjects, however, defines a difference from more standard experimental practices.

The phenomenological methods employed in NP studies derive from and simplify the methodology explicated by Husserl (1970). Varela (1996) provided the most succinct summary of the steps employed in NP research:

(1) Subjects (as well as experimenters) suspend their beliefs or theories about experience;
(2) Subjects gain intimacy with the domain of investigation by reflectively focusing on how they are experiencing the stimulus;
(3) Subjects offer descriptions that are then intersubjectively validated.

One important aspect of NP method is the avoidance of pre-defined categories. This correlates with the emphasis on first-person report and the suspension of theories. It reflects the attempt to stay with the subject’s experience without imposing external criteria or concepts, or assuming that we already know what such experience is like. In Lutz et al. (2002), for example, categories used to analyze the results were drawn exclusively from phenomenological reports developed in a preliminary set of trials. The procedure can be guided by the experimenter through open questions, that is, questions directed at the subject’s experience rather than their opinions or theories (see Pierre, 1994; Petitmengin-Peugeot, 1999). Such questions do not impose pre-determined theoretical categories. Rather than asking “Do you think this experience is like X or Y, or Z?” the experimenter asks simply, “How would you describe your experience?” By posing open questions immediately after the task Lutz et al. (2002) motivated focus on the implicit strategy used by the subject, or the degree of attention implemented during the task. The descriptive categories were then intersubjectively validated across subjects in determining the descriptive categories that were used in subsequent testing. The phenomenological categories then merit scientific validation through correlation with objective measurements of behavior and brain activity. Varela (1996) and Varela and Shear (1999) proposed a system of *dynamic reciprocal constraints* (DRCs) to illustrate this interplay of multi-source data. In the DRC model, constraints on data come, not from an externalized assumption of objectivity, but from a co-regulated interaction of subjective experiential reports and analyses of neurophysiological data. DRC distinguishes neurophenomenology from other approaches to handling first-person data by establishing a model that captures the interaction of scientist and experimental subjects.

On the one hand, in contrast to DRC, in many cognitive scientific studies first-person data will be collected in the form of pre-defined scales and surveys. Less typically, participants may be interviewed, but every participant would be exposed to identical questions. Of course, these are legitimate scientific practices, as cognitive science attempts to attribute any variation in behavior to either experimental manipulations or individual differences. Researchers seek to control the parameters to draw strong conclusions about the influence of external factors on individuals. Such studies analyze data over identical conditions applied to diverse participants, but in so doing, they risk overlooking important aspects of experience such as attentive states, emergent distractive thoughts, or individual cognitive strategies.

On the other hand, the particular NP paradigm proposed by Varela and used in the Lutz et al. (2002) study is clearly limited both in terms of the types of experiments that can be conducted and by the requirement that subjects be trained in phenomenological method. In addition its focus is on the precise relation between brain dynamics and first-person experience. Alternatively, it is also possible to introduce previously established phenomenological insights or distinctions into the design of an experiment (so-called “front-loading” phenomenology; Gallagher and Varela, 2003). This, approach, which remains consistent with neurophenomenology and the DRC model, avoids the necessity of training subjects. It also widens the scope of experiments that can be informed by phenomenology and widens the scope of the dynamic factors that can be studied to include extra-neural and extra-experiential factors, such as bodily, environmental, social, and cultural constraints.

In this same spirit of widening the scope of neurophenomenology, Petitmengin (2006) argued that there are ways to help an untrained participant accurately articulate subjective experience. The solution is a detailed interview method. Petitmengin acknowledges that it is wrong to assume that simply being aware of an experience would, necessarily, improve the articulation of the experience. She suggests that phenomenological interview techniques can help untrained participants attend to and articulate their experience. To test this idea, she conducted phenomenological interviews with nine epileptic patients over the course of eighteen months. All of the participants experienced pharma-resistant temporal lobe focal seizure epilepsy. The interviews were used to generate phenomenological clusters of preictal state experiences, which were subsequently used to interpret EEG readings in a novel manner. The results suggested that preictal experiences are manifest in epileptic patients much earlier than neuroscientists had expected (Petitmengin, 2006). While a questionnaire might have provided quantitative data that could generalize something about preictal experiences, the purpose for the phenomenological interview was to go much deeper. The interviews sought a smaller number of experiences in higher levels of unique detail. Using phenomenological interviews in these contexts, Petitmengin collected data that fed into the DRC paradigm and yielded results that contributed to both the theoretical science of epilepsy and the therapeutic know-how of patients involved in the research program. The subjects in the study were able to become aware of subtle experiential symptoms that predicted the arrival of a seizure, and were able to take countermeasures to interrupt these processes and avoid the seizure (Petitmengin, 2006).

First-person data inevitably pose large philosophical problems for empirical science, and these problems are manifest in two philosophical challenges to NP. One challenge involves reductionism, which is the proposition that explanations of consciousness and cognition can be reduced to purely physiological explanations, thereby eliminating the need to talk about first-person experience. The other challenge concerns sufficiency; it questions the capability of neurophenomenology to deliver on its promises to reveal anything that traditional scientific methods cannot capture.

One might respond to the reductionist challenge by arguing that science must strive “to explain what there is” (Gallagher, 2007, p. 311). Consequently, the category of *what there is* must not omit those things that are not quantifiable or exclude those phenomena that involve extra-neural factors (e.g., social interaction processes, which are not reducible to individual mechanisms; De Jaegher et al., 2010). Researchers ought not to accept the elimination of first-personal processes and unique experiences because of the rigidities of scientific methods; rather science can expand its methods to include the phenomenological practices that capture, investigate, and explain what may be irreducible to strictly neural processes. Precisely by refusing to objectify subjective experience, phenomenology can allow science to encompass a broader field for methodological examination. To simply accept the reductionist doctrine is to prematurely reject the potential of phenomenological experimentation.

The second challenge hinges on a narrow view of the problem to be solved. Bayne (2004) criticized neurophenomenology for failing to close the “explanatory gap” (between consciousness and purely physiological events) and offering no distinct approach for doing so. This view accepts a certain dualist and internalist way of defining the problem of consciousness, often referred to as the “hard problem” (Chalmers, 1995). On this view, consciousness (and the rich and diverse experiences of humans found in all kinds of situations) should be explained strictly in terms of brain processes, yet there seems to be no way to construct a causal bridge between purely physiological processes and first-person experience. This way of defining the problem can itself be challenged by suggesting that non-neural factors, such as extra-neural bodily processes, environmental affordances, social interactions, and cultural factors need to be part of the explanatory mix, and that there are perfectly legitimate scientific disciplines that study such phenomena. Even if one accepts the idea of an explanatory gap, however, as Thompson et al. (2005) point out, it involves different dimensions: conceptual, epistemological, and methodological.

• The *conceptual gap* involves the need for a conceptual framework to integrate first-person experience, the ability to report, and the manifestation of neurobiological responses.
• The *epistemological gap* includes issues of meta-awareness, or the potentially deforming effects of reflectively attending to experience and re-experiencing something in memory.
• The *methodological gap* involves the means by which first-person data is generated, handled, and analyzed.

Whether, as neurophenomenology matures, it can address all of these issues remains to be seen. The present work focuses on the methodological issue, but it would be erroneous to assume that any of these challenges function in isolation.

Notwithstanding these broader issues, there is reason to believe that NP methods can accomplish something important. For example, studies using phenomenological techniques in the evaluation and therapies of epilepsy and other neurophysical conditions indicate validity for the broader category of NP approaches (see, e.g., Le Van Quyen and Petitmengin, 2002). Accordingly, by focusing on some methodological issues in the following sections we propose to improve NP procedures, which can, in turn, contribute to providing a better understanding of human experience. Neurophenomenology can give us better ways of handling first-person data in a reliable and productive way, providing clarifications and classifications that are not captured by typical cognitive science approaches. However, in order for the full potential of neurophenomenology to be achieved, refining the procedure for application is necessary. We have seen developments in neurophenomenology in the past two decades, but detailed lessons learned from a case are suggested below to aid those preparing or considering using NP methodology. The following sections are proposed to serve as a practitioners guide for implementing NP methods. These recommendations are novel and explicit instructions for multidisciplinary instantiation of NP methods, but are presented with the goal of inviting continued advancement of NP methodology.

## OVERVIEW OF THE BASELINE EXPERIMENT

The preceding sections have outlined the philosophical and theoretical position of neurophenomenology, positioning it as both complementary to some, but divergent from other practices of cognitive science. In this section we introduce a baseline experiment conducted using NP methods. We reflect on a number of methodological problems that became apparent in the post-experimental evaluations, problems that have concrete methodological solutions. The baseline experiment thus provides a good opportunity to reflect on some potential issues that may arise as experimenters attempt to employ NP methods. Some of these issues may be specific to this particular kind of study; others may apply generally to NP investigations.

This baseline experiment focuses on experiences undergone by astronauts during space flights, recorded in their in-space journals and in subsequent interviews. These experiences are described variably as deeply esthetic, spiritual, or religious, and they involve affective states of awe, wonder, curiosity, and humility (AWCH). The baseline study attempted to replicate and understand these experiences by placing subjects in simulated space-travel environments, recording neurological data (using EEG and functional near-infrared spectroscopy, fNIR) and physiological data (using ECG), and correlated first-person experiences (using phenomenological interviews and psychological surveys).

### SPIRITUAL AND ESTHETIC EXPERIENCES

For the purpose of the baseline study, the researchers examined the constructs of AWCH. AWCH recur in the experiential accounts collected from astronauts’ (including cosmonauts’) journals and interviews. The astronauts’ narratives carry a unique power for many reasons. First, their demographics consist largely of scientists. Some significant number of them return from the experience of spaceflight changed, and some of their reports indicate that these changes are spiritual in nature. They report being moved affectively in a manner they describe as eliciting an internal change. They return to earth with a sense of *something bigger* “out there.” Second, they experience something that few other humans experience, extraterrestrial space. This is not to deny, of course, that there are numerous experiences that could elicit AWCH here on earth (e.g., witnessing birth, seeing natural spectacles like the Grand Canyon or Victoria Falls, or walking through icons of human ingenuity such as the Great Wall or the Coliseum).

In the past decade, scientists have promoted the cultivation of awe as a beneficial characteristic for clinical psychology and other areas of patient care. This perspective comes out of a larger movement that embraces an interdisciplinary approach to studying religious and spiritual experience more broadly. The larger project, as Taves (2010) would frame it, attempts to apply scientific method to aspects of religious texts and explain human animals’ senses of spirituality. This approach, however, is distinct from previous ones that aimed at naturalizing human spirituality (Dewey, 1991; Maslow, 1994). A standard naturalized approach would consider every aspect of a phenomenon under an objective lens. In contrast, an interdisciplinary approach that incorporates NP methods works to incorporate the potential value of subjective data in the discussions of spiritual and esthetic phenomena. This is still a naturalized approach, but not one that attempts to reduce experience to objective data. One ambitious goal for such an approach would be to investigate the relationships between affect, reason, and spiritual experiences in an empirical manner (as opposed to leaving such inquiries just to philosophical or (heaven forbid!) theological speculation). Ultimately, interdisciplinary research, such as that conducted through neurophenomenology, helps mend disparate fragments of the dynamic complexity that links the human mind and human behaviors in experiences of all sorts.

To explore the interdisciplinary challenges inherent to NP examinations of spiritual and esthetic experiences in any useful way, it is essential to narrow the focus and establish a shared nomenclature. In the research examined herein, awe and wonder provide construct exemplars. Awe, as a component of spiritual and religious experience, has been discussed in historic philosophical traditions. In the eighteenth century, Edmund Burke’s philosophical treatises struggle with the sublime and beautiful, binding these constructs conceptually with awe-filled emotions. These emotions do not imply pleasure, in its most obvious sense, but Burke (2010) asserts that people experience awe inseparably from terror, power, obscurity, and humility.

For purposes of our study, Gallagher et al. (in press) define *awe* as a *direct* and *initial feeling* when faced with something incomprehensible or sublime. In contrast to the directness of awe, *wonder* demands cognitive reflection. Fuller (2009) argued that wonder bridges emotion with the desire to apply order to the universe. This repeats the sentiment of Magnus (1988, p. 557), who a millennium ago stated, “⋯ wonder is the movement of the man who does not know on his way to finding out.” The present research defines wonder as a more reflective feeling one has when unable to put things into a familiar conceptual framework. *Curiosity*, also involves a desire to piece things together, but in a different way. Curiosity involves wanting to know, see, experience, and/or understand *more* (Gallagher et al., in press). The object of this wanting may be technical, logical, moral, or existential. In 1914, John Milton McIndoo asserted that curiosity opposes the impulse to flee in fear. That which may incite fear at first, may become intriguing, as familiarity grows. In this respect, curiosity, which is “world-oriented,” acts as an important contrast to *humility*, which is “self-oriented.” Philosophers and theorists vary greatly concerning the nature of humility, attaching it to everything from psychological concerns of self-esteem, roles within cultures, and the limits of knowledge within the universe (Tangney, 2000). Regardless, humility demands a sense of perspective, where one must place oneself in scale to someone or something else. In the present study, humility is a sense one has about one’s relation to the universe (an issue of scale) or one’s significance (an issue of moral aspect). Starting with these working definitions of AWCH, researchers applying an interdisciplinary NP approach can examine experiential phenomena and offer possible interpretations of the findings.

### THE ART AND SCIENCE OF SIMULATION

The baseline experiment was part of a collaborative interdisciplinary project entitled *Space, Science, and Spirituality*. Researchers from multiple disciplines coordinated their efforts to create an immersive simulation of space travel for the purpose of gathering neurophysiological and phenomenological data. Astronauts had captured the novelty of space travel in written journals containing detailed descriptions of both visual and affective experiences. Hermeneutic and semantic analyses were used to identify and categorize the ways astronauts verbally expressed their experiences in these texts. Details from these accounts then informed the design of a mixed-reality immersive test environment where participants viewed simulations of space views, and provided a narrative comparison for first-person experiential accounts. Simulation artists and engineers used NASA images selected on criteria developed by researchers at the *Kolleg-Forschergruppe Bildakt und Verkörperung* (Humboldt University, Berlin). The *Bildakt* analyses revealed necessary requirements for the images used in the simulation including characteristics of photograph quality and image angle and perspective. With these conditions in mind, the team designed a set for a mixed-reality simulation wherein participants were immersed in the sounds and visuals resembling something like a science fiction movie set.

Both the baseline study and other experiments in this project used simulation technologies to generate controlled conditions. Varying degrees of realistic experiences are possible within virtual reality simulators and they can allow for NP research to be conducted with high levels of control (Sherman and Craig, 2002). To establish an effective simulation experience, designers must address immersion, point-of-view, and practical venue concerns. Thus, considerations of quality and presence should be integrated into the simulation design. That is, the simulation should support a willing suspension of disbelief and in doing so allow for a narrative transfer that takes the participant into the world presented within the simulation. The image requirements from the *Bildakt* analyses (listed above) reflect an intention to increase immersion, clarify point-of-view, and be applicable to the limitations of the mixed-reality simulation environment used in the experiment.

Aspects of context were incorporated into the simulation design and were crucial in the experimental strategy. All participants experienced identical “launch” context narratives and were placed in the same physical environment. Although the contextual cues for all participants were objectively identical, the experienced context varied due to a variety of factors, including the participant’s background and their current bodily state. In all research, contextual circumstances, both objective and subjective, contribute to the experience. Context consists of any information or factor that can be used to characterize the circumstances or situation of the participant. Multiple factors might be relevant to the interaction between a participant and the simulation and may or may not be manifest within the simulation itself; in this regard, the participant determines the perception and experience of relevancy. In basic NP research, this means that control of contextual variables within the experimental environment is of utmost importance.

The baseline study applied these principles of simulation context design in the development of the mixed-reality simulator and the conditions presented therein. The experimenters presented the study to participants in terms of simulated space travel and worked to support the narrative that reinforced the idea that the participant had been selected to have the unique opportunity for virtual space travel. The experimenters worked to maintain the story narrative of an impending launch, even while placing neurophysiological sensors on the participant.

Researchers collected real-time physiological and neurophysiological measurements using EEG, ECG, and fNIR while the participants were immersed in the mixed-reality environment and observed the space scenes. The experimenter sat unseen by the participant, outside of the space vehicle and could only be contacted through radio control.

After “suiting up” with the various physiological and neurophysiological monitors, the participant was in the mixed-reality environment which resembled the interior of a space vehicle (based on images of the interior of the International Space Station). The participant then, after a short delay, experienced a launch sequence (countdown and a convincing audio experience, as the chair and space vehicle were stationary), followed by silence. Once the participant was “in space” large computer monitors embedded in the walls of the space vehicle opened virtual portals revealing dynamic images of space, including views of Earth, Moon, the International Space Station, and expanses of stars. After the participant viewed the images, the space vehicle “returned to earth” (relayed to the participant through radio control) and the participant answered brief questionnaires, repeating some of the questions that had been asked previously and introducing questions of workload. Experimenters removed all sensors and then brought the participant to meet an interviewer. The interviewers were philosophy graduate students trained in phenomenological interview techniques. Interviewers escorted participants to another area for interviewing, with the succinct goal of exploring the participant’s experience during the simulated space flight.

From the beginning to the end, the baseline experiment was executed much like a relay race, with researchers passing the baton from one stage of the experiment to the next. The analysis of the collected data required a division of labor by the various contributing disciplines. Human factors psychology and neuroscience experts examined the data from the psychological and neurophysiological measurements. Phenomenologists reviewed the transcripts and recordings of the interviews and conducted linguistic and hermeneutical analyses. The latter was informed by a previous analysis of the astronaut journals that provided 37 categories reflecting AWCH experiences. That starting point was leveraged for a comparative evaluation of the participant interviews. The results showed promise. For example, in preliminary analyses, the results of the neurophysiological data indicated engagement of frontal lobes during the feeling of wonder and parietal lobes during physical affect. Participants who reported experiencing awe, wonder, religiousness, or spirituality were compared to those who did not indicate such experiences. Neural activity varied significantly between experiencers and non-experiencers and was greater for the Earth view than the Deep Space view. In addition, participants who reported higher levels of religiousness (as indicated in the questionnaires) were more likely to report awe and wonder when viewing the Earth (as opposed to only stars).

## LESSONS LEARNED

The results did not lack merit. To the contrary, the results provided information about human phenomena that otherwise have garnered little empirical exploration to date. The results of the baseline experiment contribute to a compelling case in favor of further exploration using neurophenomenology. The work supports the application of open-ended interviews for a broader range of basic-research contexts. However, the experiment provides “lessons learned” for improving NP methods in hopes of generating more conclusive results.

### LESSON #1

*Neurophenomenological experimentation must purposefully and systematically include the creation and maintenance of shared mental model (SMM)*.

Neurophenomenological research must embrace systematic and thorough creation of SMMs as part of the neurophenomenology as applied philosophy in the research world. Its intrinsically interdisciplinary nature demands that contributing domain experts avoid the “passing the baton” approach that can result in a mere collection of data in a non-contextualized way (Van Rijnsoever and Hessels, 2011). Each part of the project (in this case, analysis of texts, analysis of images, construction of the simulation, neurological data collection, questionnaire administration, phenomenological interview) has to be understood by all members of the team in terms of the whole (Bockelman Morrow and Fiore, 2013). As described in the previous section, our team for the baseline experiment consisted of human factors psychologists, philosophers, art historians, astronauts, simulation developers, and computer engineers. Each contributing member adds value, but entered the collaboration with differing vocabulary or semantics for concepts and constructs. Working culture for each was substantially different, yet understandable for the given domain. According to expertise, each allocates priorities of tasks differently. The NP approach can only be accomplished with all contributing members operating from a shared lexicon and conceptual framework. To achieve that, specific techniques for accomplishing the SMM vary and should be considered according to the group and its circumstances (Cannon-Bowers et al., 1993). In preparation of another round of studies based on the preliminary findings of the baseline experiment, the following techniques have been employed: concept mapping prior to experimentation, ongoing training of researchers regarding NP theories and methods, and preparing analysis to handle the complexities inherent to the NP data. These are examples of techniques for addressing the needed SMM. Logistically, these take time. Scheduling regular meetings for brainstorming and teaching, as well as creating a glossary and living conceptual framework document are simple practices and tools for successful NP implementation. The outcome should not only be a productive experimental development collaboration, but also lead to synthesized results, not individualistic pieces in interpretation from each domain’s perspective. Instead the results are the whole or a big chunk of puzzle.

### LESSON #2

Neurophenomenological experimentation should be held to the high standards of experimental design and execution to achieve variable control, reliability, generalizability, and replication of results.

In the complexities of carrying out an interdisciplinary study with multiple parts, problems can arise. In the baseline study, there were many possible variables that could have explained components of the experience (e.g., launch narrative, the mixed-reality components of the simulator, and changes in setting between experience and interview). It was therefore impossible to conclusively determine which manipulations generated the various aspects of the experiences. In part, this problem in the baseline study was the result of inadequate SMMs mentioned above. Team members, working within the confines of their own specialized disciplines were not always able to see the whole picture, and this had an effect on the overall design of the experiment. This, of course, is not inevitable, or necessarily a characteristic of other NP experiments, but that this problem did characterize the baseline study suggests that adopting different practices is something that needs to be made explicit. Accordingly, the second methodological lesson is a reminder that psychology and the cognitive sciences already have a time-tested tradition of precision in experimentation and that neurophenomenology can benefit from attending to many of the practices involved in this tradition.

As researchers design a NP experiment, they should consider many of the questions that their counterparts in traditional cognitive science might ask. For example, does it make sense to generalize first-person data? Cognitive scientists, with a firm footing in psychology, consider the extent to which any finding can be generalized to the population at large, and that consideration may affect the manner in which the data is both collected and handled. Neurophenomenologists need to grapple with that question as well, and avoid oversimplification of the factors that contribute to the results.

In addition to generalizability, cognitive psychology also considers verification. A well-formed NP study needs to consider procedures for the verification of subjective experience. The baseline study, due to the length of experimental sessions and the uniqueness of using the NP approach, only included one aspect from each field that compose neurophenomenology. In other words, neurological and physiological measures from neuroscience were used to assess one part of the participants’ experience (physical response), questionnaires from psychology were used to assess another part of the participants’ experience (demographic information, traits, and cognitive response to the immersive environment), and phenomenological interviews from philosophy assess participants’ linguistic attribution of their experience in the environment as though they were reliving the experience. There was no overlap; no checks and balances. Strictly, this was a neurophenomenology study, but not an optimal application of NP methods, which thereby limit the power of the interpretation of the results. Again, to iterate, the data attained from the baseline experiment is useful and informative to the phenomenon under investigation as seen from each discipline, but perhaps short of the ideal of neurophenomenology.

Depending on the study, other disciplines can provide complementary tools for verification. On its own, a Likert scale of self-reported affect (a tool from psychology) will not capture lived experience. However, it can provide correlations and comparisons that can provide verification when interpreting the findings from the phenomenological interviews. When designing the experiment, researchers can incorporate established methods from psychology, taking care to avoid influencing the phenomenological interview. For example, a sliding qualitative scale of affect can be given after the phenomenological interview. The information from the scale can provide support to the textual analyses of the interviews. The scale cannot, on its own, capture unique lived experience, but it can add credence to the basic findings in neurophenomenology.

In regard to using methods from cognitive science, researchers designing NP experiments must also consider the replicability of their experiments. For example, neurophenomenology has been used to explore experience in epileptic preictal states (Le Van Quyen and Petitmengin, 2002, Petitmengin, 2010). In this example of applied neurophenomenology, the lack of control (variation between subjects) provides an explanation for any inability to reproduce results. After all, the patients were not being studied to capture generalizable aspects of preictal experience, but to improve the identification of preictal states in epileptic patients. The study not only made a significant contribution to epilepsy research, but it has also played an immeasurably valuable role in the lives of patients who benefited directly from participating in the study. However, basic research should typically include high levels of control and produce highly predictable and reproducible results and this can also be accomplished in NP methods. The baseline study does hold to the methodological rigor for reliability. However, this lesson learned is from the literature foundation from which we derived our phenomenological interview practices. One of the key objectives of neurophenomenology is to function within experimentation. Therefore, attention must be paid to experimental rigor, precedence, and scientific acceptance. This understanding benefits the ability of a multidisciplinary team to succeed in employing the NP approach.

To do this, the experimental design might include the embracing of simulation technology. Simulation test beds allow for high variable control and precise stimulus/response recordings, consequently increasing successful replication. Simulations can be shared between institutions, permitting more diverse population testing (and bringing the results higher generalizability as well). For example, future experiments in our project will use a portable simulation environment. This form of immersive simulation allows the presentation to be packed up and taken to another location, so that other locales can benefit from the technique. It also is a straightforward, and relatively cost-effective form of simulation presentation, so that laboratories with limited simulator resources can erect similar systems. A digitally controlled presentation of experimental stimuli allows for improved generalizability, replicability, and verification, and can thereby give more credence and credibility to the NP findings. Interplay between the phenomenological project and some techniques of cognitive science can generate a unique integrative methodology. As such, neurophenomenology should take the best of the practices of cognitive science, while contributing its unique techniques that have not been a common part of the cognitive science toolbox.

### LESSON #3

The phenomenological interview places the impetus for training on the interviewer, not the participant, so that the interviewer may act to support the participant in precise experiential reporting.

The third lesson to extract from the baseline study is that the real question of phenomenological training should shift from focusing on the participant to the interviewer.

Though Lesson #2 argued for the importance of adopting specific practices from cognitive science, Lesson #3 involves the aspect of neurophenomenology that stands in contrast to cognitive science. To work through the argument, it is essential to first describe the role of training in the interview. The “training trade-off” depicts how the line between the interviewer and interviewee can dissolve, so that the interviewer actually *participates in* the reflection and articulation of experience. This participation on the part of the interviewer is a marked difference from the objective techniques of cognitive science and as such, has potential for opening forms of data collection and analyses of experience that have not yet been fully explored.

As discussed above, much NP research has been based on the idea that only a person trained in introspective or phenomenological techniques can provide details with the degree of precision required for meaningful results (cf. Varela and Shear, 1999; Lutz et al., 2007). Petitmengin’s (2006) technique strays from that position and accommodates information from the untrained participant. In the baseline experiment and further continuing experiments, we have adhered to the interview techniques found in Petitmengin (2006). One of the observations made from the baseline experiment is that this is not a training binary (i.e., participants either are capable of good phenomenological reflection or completely lack self-awareness), but a continuum. This observation was attained in reviewing the interview transcripts. The questions asked directly influenced the articulations from the participants and the follow-up paraphrasing or reflecting likewise affected the topics discussed by the participants. The level of phenomenological (or introspection or mindfulness) training on the part of the participant leads to a “training trade-off” in respect to the interviewer. The less experienced the participant (to the limit of never having practiced phenomenological reflection), the more training the interviewer needs in ways to help draw out the experience while carefully avoiding any priming of the participant. The opposite is true as well; a participant well-trained in introspection, reflection, or mindfulness might not require a highly skilled interviewer to draw out the experiential data.

The impact on methodology is that the emphasis shifts from a question about the degree of training a participant does or does not receive. This degree of self-awareness cannot be controlled in participants with the interview approach. Instead, the onus for training is directed toward the interviewer. The training trade-off is compensatory, in that the interviewer’s skill will improve the chances of the untrained participant’s successful articulation of her experience. If the interviewer is working with Buddhist monks, she may not need to receive a great deal of training and may be able to simply tell the participant the focus of the study. Conversely, if the same interviewer is working with undergrads at any given university in the West, she may need to pull from a collection of tools and techniques to give the participant the capacity to access the thoughts and feelings experienced.

It should be noted that, while the training trade-off is presented, primarily as a methodological lesson, it has important theoretical weight as well. This dynamic between interviewer and participant has value for the debate regarding phenomenological experimentation. The dynamic interaction demanded by the NP interview method is not part of traditional cognitive science. In the NP interview, there is a cognitive off-loading by the participant onto the interviewer that traditional psychology does not always recognize. The phenomenological interviewer has the power, if executed in the manner described herein, to do some of the cognitive work of focusing the participant precisely on the participant’s lived experience, a task that may be otherwise impossible for the untrained participant. This is not one of the tools of traditional cognitive psychology. When psychologists employ the similar technique of introspection (perhaps in therapeutic sessions like hypnosis), they involve actually training the participant to look inward. The goal, in such cases, is not to extrapolate a description of an experience itself. Rather, interview questions are tools used, perhaps by applied psychologists/therapists, but certainly not typically by basic researchers. When neurophenomenology employs the interview, it is a core method that is fundamentally part of the experimental approach, and as such it changes the way the first-person experiential data is captured and handled.

This does not degrade the first-person experience, treating it as third-person data (as Dennett, 1991 suggests science requires). Instead, the interviewer actually engages (in a second-person way) to preserve the first-person experience. The goal of the interviewer is not to remain an objective third-person observer (an important aspect of cognitive science methods); the goal is to assist the participant in “opening” to her own experience. The interviewer can do the cognitive work of keeping the reflection on target, exploring the experienced thoughts, emotions, and sensations of the participant, as the participant would do on her own if she were so trained. With properly trained interviewers, the participant need not be trained, as the interviewer adopts the cognitive burden for making the reflective attitude possible. The interviewer is essentially doing a share of the processing, interactively working to assist the participant in accessing her own experiences. In this process, there is no need for the interviewer to ascribe any mental states to the participant, or to engage in mindreading (understood in the standard way in social cognition), nor is there a need for the interviewer to access her own mental models or simulations of what the other’s mind is. The interviewer, quite plainly, participates, interactively, in the articulation of the participant’s experience. In this way, the scientific concept of bias is important to eliminate. This requires interviewers to know the scientific method and need for controlling extraneous variables. Language mediates (with support of second-person pronouns used by the interviewer), to direct the naïve participant toward accessing the embodied memory of experience, just as experience in mindfulness training would allow the expert meditator to access the experience independently. Traditional cognitive science often tries to force the participant into an objective mold of predesigned conceptual frameworks. In contrast, the interactions of the interview allow a switch of directions altogether and temporarily allows the researcher to participate in the participant’s subjectivity. The resulting interview can make manifest an experiential record that traditional methods cannot reveal.

Consequently, the third lesson involves a “training trade-off” in integration of the interview into experimental design and execution. NP methods can work with this adjustment in focus from the participant to the interviewer, and the re-evaluation needed for each unique experiment. The interviewers can pull from multiple tools and techniques with the aim of eliciting the necessary acts of reflection and articulation. Petitmengin (2006) outlines many of the practical techniques, but in the contexts of specific experiments one has to consider how such techniques can be put to use. Interviewers should be subjected to rigorous training and rehearsal to prepare them for the interview tasks. This training should include an assessment of the anticipated personalities and behavioral idiosyncrasies likely to be expected by a given population of participants. That assessment would direct the focus of the training, so that interviewer responses to anticipated barriers would become more natural and automatic. Training should also include mitigation of interviewer personality quirks that might otherwise influence interactions. The baseline study interview transcripts revealed that one interviewer was more extroverted and used more informal language than the other two interviewers, which seemed to elicit different responses perhaps because of perceived rapport by the participants. The interviewer should behave as if she is a prosthetic for the participant and the questions must support that participant’s exploration of her experience without imposition of the interviewer’s bias. In this way, the interviewer is an extension of the scientific team members involved in a NP experiment.

## CONCLUSION

Reflecting on our own experiences in conducting a baseline study employing NP methods, we have explicated a number of problems that we encountered and made suggestions for addressing such problems. The first involves improving communication practices among research team members and establishing a shared understanding of the methods and goals of neurophenomenology. This is especially important since neurophenomenology involves certain ways of rethinking received scientific procedures and is not the standard approach in which many of the researchers have been trained. The second involves the importance of incorporating some of the best scientific practices into NP methods, especially those that involve control, reliability, generalizability, and replication. The third suggestion involves the use of a phenomenological interview technique and the question of who gets trained, and how. Each of these suggestions should be considered in the variable contexts of NP research.

## Conflict of Interest Statement

The authors declare that the research was conducted in the absence of any commercial or financial relationships that could be construed as a potential conflict of interest.
